# Transcriptomic data meta-analysis reveals common and injury model specific gene expression changes in the regenerating zebrafish heart

**DOI:** 10.1038/s41598-023-32272-6

**Published:** 2023-04-03

**Authors:** Marius Alexandru Botos, Prateek Arora, Panagiotis Chouvardas, Nadia Mercader

**Affiliations:** 1 Institute of Anatomy, University of Bern, 3012 Bern, Switzerland; 2 Department for Biomedical Research, University of Bern, 3012 Bern, Switzerland; 3 Department of Urology, Inselspital, Bern University Hospital, 3010 Bern, Switzerland; 4 Centro Nacional de Investigaciones Cardiovasculares CNIC, 28029 Madrid, Spain; 5 Present Address: Laboratory of Systems Biology and Genetics, Institute of Bioengineering, School of Life Sciences, École Polytechnique Fédérale de Lausanne (EPFL), Lausanne, Switzerland; 6 Present Address: Swiss Institute of Bioinformatics (SIB), Lausanne, Switzerland

**Keywords:** Gene regulatory networks, Transcriptomics, Regeneration, Cellular signalling networks, RNA sequencing, Transcriptomics, Gene expression profiling, Systems analysis

## Abstract

Zebrafish have the capacity to fully regenerate the heart after an injury, which lies in sharp contrast to the irreversible loss of cardiomyocytes after a myocardial infarction in humans. Transcriptomics analysis has contributed to dissect underlying signaling pathways and gene regulatory networks in the zebrafish heart regeneration process. This process has been studied in response to different types of injuries namely: ventricular resection, ventricular cryoinjury, and genetic ablation of cardiomyocytes. However, there exists no database to compare injury specific and core cardiac regeneration responses. Here, we present a meta-analysis of transcriptomic data of regenerating zebrafish hearts in response to these three injury models at 7 days post injury (7dpi). We reanalyzed 36 samples and analyzed the differentially expressed genes (DEG) followed by downstream Gene Ontology Biological Processes (GO:BP) analysis. We found that the three injury models share a common core of DEG encompassing genes involved in cell proliferation, the Wnt signaling pathway and genes that are enriched in fibroblasts. We also found injury-specific gene signatures for resection and genetic ablation, and to a lower extent the cryoinjury model. Finally, we present our data in a user-friendly web interface that displays gene expression signatures across different injury types and highlights the importance to consider injury-specific gene regulatory networks when interpreting the results related to cardiac regeneration in the zebrafish. The analysis is freely available at: https://mybinder.org/v2/gh/MercaderLabAnatomy/PUB_Botos_et_al_2022_shinyapp_binder/HEAD?urlpath=shiny/bus-dashboard/.

## Introduction

Heart failure induced as a consequence of myocardial infarction (MI) still represents a leading cause of death worldwide^1,2^. After a myocardial infarction (MI), millions of cardiomyocytes are replaced by an irreversible fibrotic scar. In contrast, some species can naturally fully recover from a myocardial insult. This is the case for *Danio rerio*, the zebrafish. After a cardiac lesion, zebrafish undergo an inflammatory response and a concomitant proliferative response of different cell types, among them cardiomyocytes, that allows to regrowth the lost tissue^3^.

Different injury models were developed to simulate a MI in the zebrafish and study the elicited regenerative response. The first to be established and classical injury method is ventricular resection^4,5^. In this model, the apex of the ventricle is amputated using microdissection scissors, leading to the loss of around 20% of the whole ventricle. A blood clot is formed rapidly after surgery, followed by the formation of an epicardial cover, immune cell infiltration and proliferation of endocardial cells. At 7 days post-amputation (dpa), cardiomyocyte proliferation peaks, and at 60 dpa the heart has fully regenerated. While resection is based on tissue removal, the cryoinjury model induces tissue damage followed by replacement with newly formed tissue^6–8^. Cryocauterization is performed by exposing the heart and contacting the ventricular surface with a metal filament that is precooled in liquid nitrogen and freezes the surroundings of the contacting point. A massive but transient fibrotic response characterizes this injury model. During the first week after injury, fibroblasts accumulate at the site of injury and start depositing extracellular matrix (ECM). ECM is subsequently degraded and replaced by cardiomyocytes, that peak in their proliferation also around 7 days post-injury (dpi). Complete regeneration takes longer than after resection, and complete fibrotic tissue regression is observed around 100–130 dpi. A third commonly used injury model is based on a transgenic approach. Cardiomyocytes are engineered to express either diphtheria toxin A (DTA)^9^ or nitroreductase (NTR)^10^. DTA expression induces cell death and nitroreductase metabolizes Metronidazole into a cytotoxic agent when administered to the fish tank water. With this approach, specific loss of cardiomyocytes can be induced, without eliminating the rest of cardiac cell populations. Genetic ablation of cardiomyocytes also leads to a rapid epicardial and endocardial response and immune cell infiltration, as documented for the other two injury models. Cardiomyocyte proliferation also peaks at 7 dpi and full regeneration is achieved at 30 dpi. Genetic ablation of cardiomyocytes does, however, not induce a strong fibrotic response^9^ as observed after cryoinjury and, to a lesser extent, after resection.

RNA sequencing (RNA-seq) technology has become the standard technology for transcriptomics^11^. Since it has also been extensively used to study heart regeneration, we reasoned that publicly available RNA-seq datasets might allow to understand commonalities and injury-specific differences between the gene regulatory networks controlling heart regeneration. Use of previously generated dataset would also allow to reduce the number of animal experiments to be performed to answer this pertinent question. We therefore decided to perform a meta-analysis to study the transcriptomic differences between the different zebrafish heart injury models. Re-analysis of batch corrected datasets collected at the key time point of 7 dpi allowed to determine a common gene expression signature among all different models, but also revealed that injury models lead to a specific gene signature. We also present our data in a web interface to show our results of injury specific and regeneration specific responses.

## Results

### Batch correction according to sequencing platform allows normalization of RNA-seq data sets from regenerating zebrafish hearts

The RNA-seq data used for our analysis was retrieved from published studies and are publicly available at NCBI Gene Expression Omnibus (GEO). To find and select the studies, a literature review was performed using NCBI PubMed keyword queries. In total we used 36 samples distributed in 7 datasets for this meta-analysis (Table 1).

**Table 1:** RNA-seq Dataset of 7 days postinjury (dpi) hearts used for the meta-analysis.

Condition	Study	Dataset	Tissue	Platform	Library	Length	Extraction	Sample size	Pool	References
Sham		GSE100892	Ventricle	NextSeq 500	SingleEnd	76	polyA	4	3/4	^12^
Cryoinjury		GSE100892	Ventricle	NextSeq 500	SingleEnd	76	polyA	4	3/4	^12^
Sham		GSE112452	Heart	BGISEQ-500	SingleEnd	50	polyA	3	1	^13^
Cryoinjury		GSE112452	Heart	BGISEQ-500	SingleEnd	50	polyA	3	1	^13^
Resection		GSE129499	Ventricle	HiSeq X Ten	PairedEnd	150	totalRNA	3	6	^14^
Sham		GSE157170	Ventricle	NovaSeq 6000	PairedEnd	150	totalRNA	3	1	^15^
Resection		GSE157170	Ventricle	NovaSeq 6000	PairedEnd	150	totalRNA	3	1	^15^
Uninjured		GSE144831	Ventricle	HiSeq 2000	PairedEnd	150	polyA	3	6/8	^16^
Ablation		GSE146859	Ventricle	BGISEQ-500	SingleEnd	50	totalRNA	3	5	^17^
Uninjured		GSE146859	Ventricle	BGISEQ-500	SingleEnd	50	totalRNA	3	5	^17^
Ablation		GSE75894	Ventricle	Genome Analyzer II	SingleEnd	50	polyA	2	10	^18^
Uninjured		GSE75894	Ventricle	Genome Analyzer II	SingleEnd	50	polyA	2	10	^18^

NCBI GEO database (https://www.ncbi.nlm.nih.gov/geo/) was queried for RNA-seq studies using different heart injury models in zebrafish at 7 dpi. Metadata is presented in the table columns. **Condition**: defines the heart injury model used for this dataset, uninjured hearts, and sham procedure (mock chest surgery). **Study**: shapes are representing the datasets lab of origin. **Dataset**: GEO IDs to track the sample. **Tissue**: biological part used for the sequencing, full heart, or ventricle. **Platform**: specifies the sequencing machine used for the experiment. **Library**: sequencing library used in the experiment consisting of single or pair ended reads. **Length**: defines the sequencing read lengths. Extraction: protocol used to enrich the mRNAs, oligodT or totalRNA kits. **Pool**: number of hearts or ventricles that a sample is composed of.

**Figure 1:**
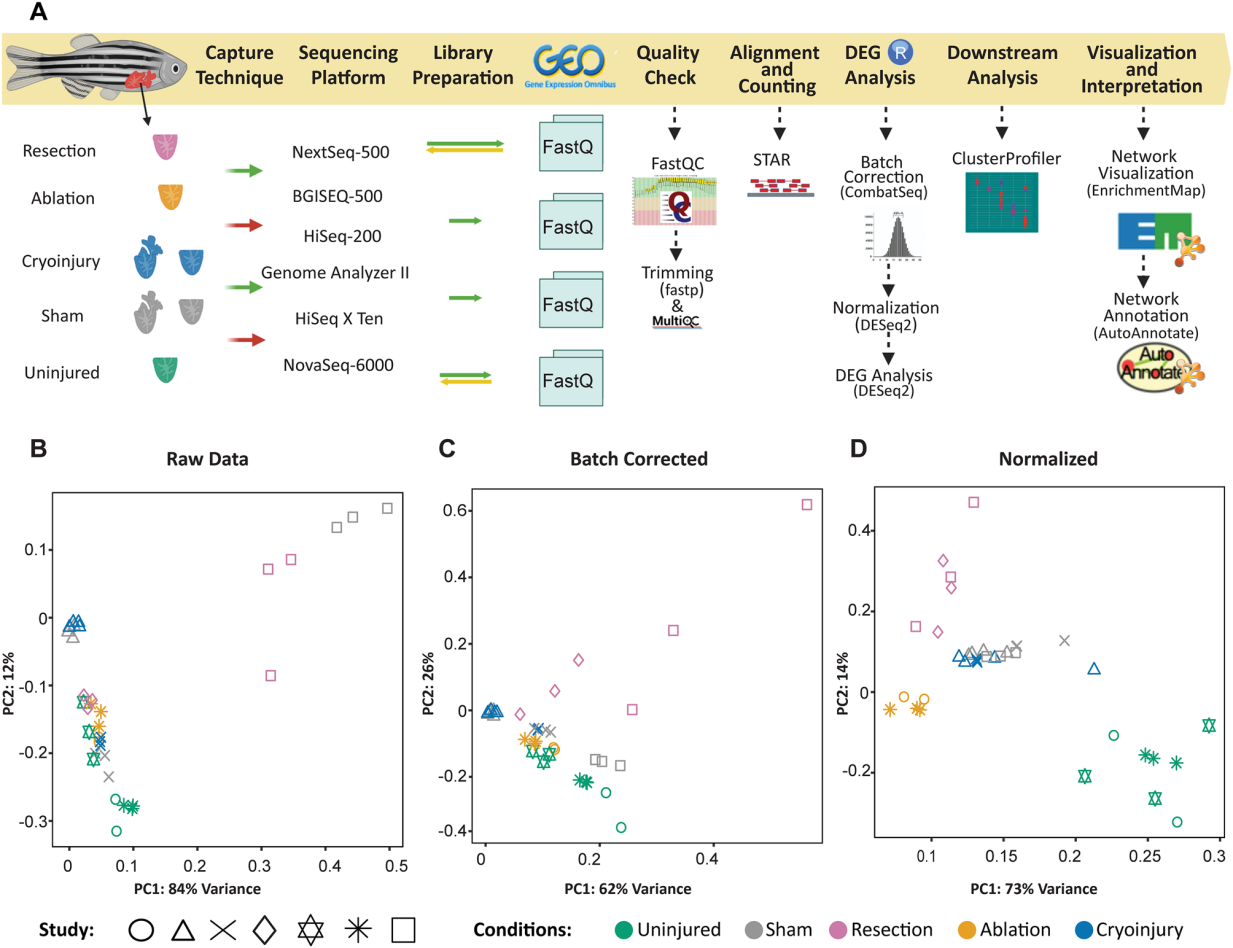
Transcriptomic meta-analysis of the cardiac regenerative response upon distinct types of injuries. (**A**) Workflow of the process used to analyze the data. Selected datasets differed in conditions of the transcriptomic data such as the capture techniques, sequencing platforms and reads structure. Data were downloaded from GEO and the processing steps to find the differentially expressed genes were performed. This was followed by downstream analysis to interpret the results. (**B**) PCA plots of the raw data before any processing. (**C**) Batch corrected, after removing the platform sequencing variable giving the highest batch effect. (**D**) DESeq2 normalized data.

The selected datasets differed in several features such as the part of the heart collected (whole heart or ventricle), the RNA extraction protocols (e.g., use of oligodT or hexamers for mRNA purification), library preparation, single or pair-end sequencing protocol and sequencing platforms being used (Fig. 1A). All these features were presumed to lead to batch effects.

Indeed, reanalyzing the 36 samples revealed the expected batch effects, as observed in the samples distribution when plotting the raw data in the PCAs. In this case we observed that the clustering of the different samples was not driven by the biological condition of the injury type but rather by the origin of the dataset (Fig. 1B).

We therefore used Combat-Seq^19^, to correct for the batch effects before we proceeded with data analysis. To correct for the batch effects, we made several attempts considering unique features that could counteract for the variability, including sequencing library preparation, length of the reads and laboratory of origin (Supplementary Fig. S1). We found that the highest data variability could be explained by the sequencing platform used. When using this feature for batch correction the data segregated clearly according to the injury conditions rather than any of the technical effects (Fig. 1C).

Post batch correction, we performed differential gene expression analysis, for which two main tools were considered: edgeR^20^ and DESeq2^21^ (Fig. 1D). With both tools, we normalized the data to remove inter-sample variability. After normalization, the data clusters reflected the injury conditions even better than just after batch correction.

### Common and injury-specific gene signatures are detected in regenerating zebrafish hearts

**Figure 2:**
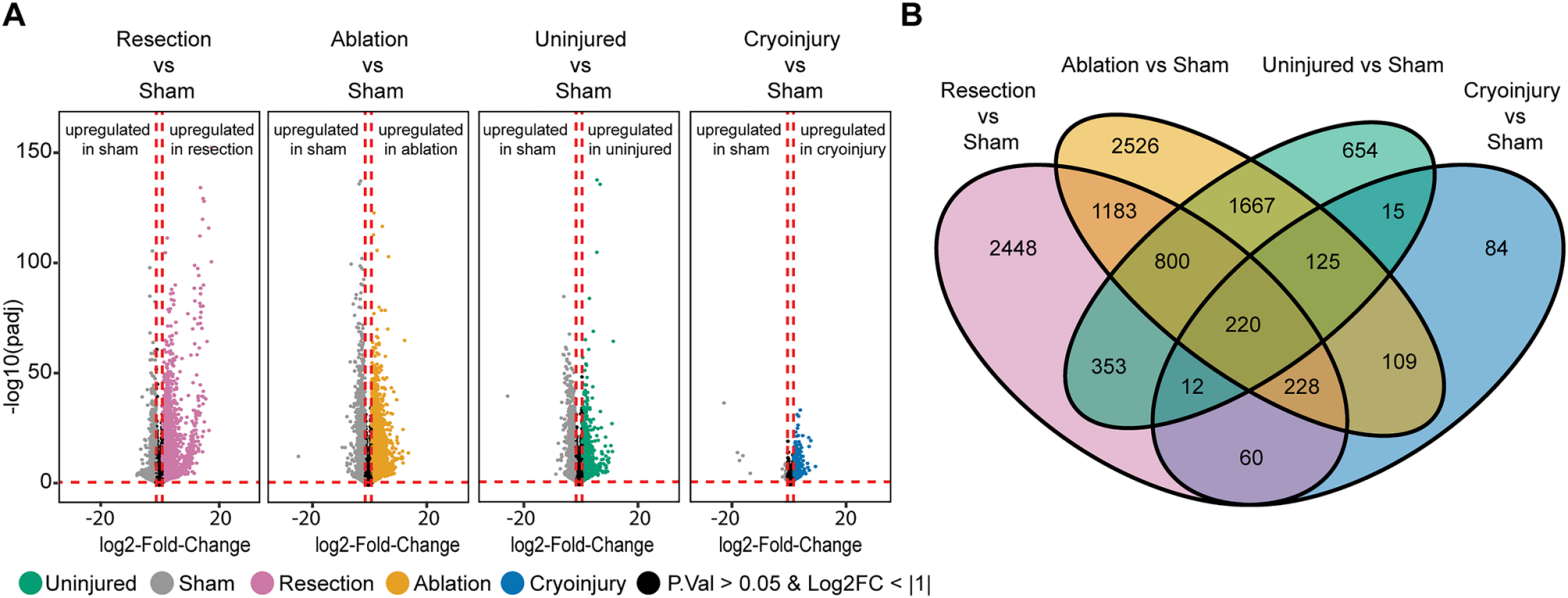
Unique and common differentially expressed genes (DEGs) in the 7 dpi regenerating zebrafish heart after resection, cryoinjury, or genetic ablation. (**A**) Volcano plot of the different conditions showing the DEGs. Grey, sham; orange, genetic ablation; magenta, ventricular resection; green, uninjured; blue, cryoinjury. Black area stands for non-significant genes with an adjusted *p* value > 0.05 or a log2FoldChange value > − 1 and <  + 1 which were not considered for the analysis. (**B**) Venn diagram of the zebrafish DEGs converted to the respective mouse orthologs, for each condition.

The normalized data was used to perform pairwise comparisons between the different injury models. The following conditions were compared: resection vs sham, ablation vs sham and cryoinjury vs sham. We also performed the comparison between the uninjured and sham-operated hearts to identify a possible systemic response elicited by surgery itself. We set a threshold of absolute log 2-fold change > 1 and adjusted *p* value < 0.05. After an exhaustive comparison of the differentially expressed genes (DEGs) resulting from DESeq2 as well as edgeR analysis (Supplementary Table S1), we found that the list of DEGs from both analysis workflows overlapped to a great extent (Supplementary Fig. S1). DEG calling was more restrictive for DESeq2, as most of the DEGs within the DESeq2 analysis were a subset of the DEGs obtained from edgeR analysis. Thus, to keep the analysis restrictive, we selected the DESeq2 dataset. The comparison ablation vs sham revealed the greatest number of differentially expressed genes (DEGs) (n = 6858), followed by resection vs sham with n = 5304 DEGs. Surprisingly, we observed more DEGs (n = 3846) when comparing uninjured vs sham condition, than in the comparison of cryoinjury vs sham (n = 853) (Fig. 2A).

We reasoned that all injury models consist of two responses: a response that would be specific to each type of lesion and a regeneration response expected to be conserved across injury models. To find the injury-specific responses we focused on the genes that are not shared among injury conditions but are unique to the injury method (Fig. 2B and Supplementary Fig. S1). We found the highest number of unique DEGs related to the response elicited by genetic ablation of cardiomyocytes (ablation vs sham; n = 2526 DEGs). This was closely followed by the comparison of resection of the ventricular apex of the heart vs sham operation, with (n = 2448) unique DEGs. Unexpectedly, only (n = 84) genes were uniquely differentially expressed upon cryoinjury when compared with a sham surgery (cryoinjury vs sham). This number was even smaller than that of DEGs specific to the comparison of the control groups uninjured vs sham (n = 654).

### Network integration of biological processes enriched in the different injury models

For a better understanding of the functions and processes associated with core regeneration and injury-specific gene sets we used Gene Ontology (GO) annotations for downstream analysis. Since GOs are poorly annotated in zebrafish but well annotated in mouse, we converted these zebrafish genes into Mouse EntrezIDs (Supplementary Table S2).

We rationalized that even though we performed the overrepresentation analysis using the unique genes across the injury models they might still be part of the same biological processes. We therefore used the mouse orthologues and clusterProfiler^22^ for downstream analysis of the GO Terms to investigate the overrepresented Biological Processes (BP) (Supplementary Table S3).

**Figure 3:**
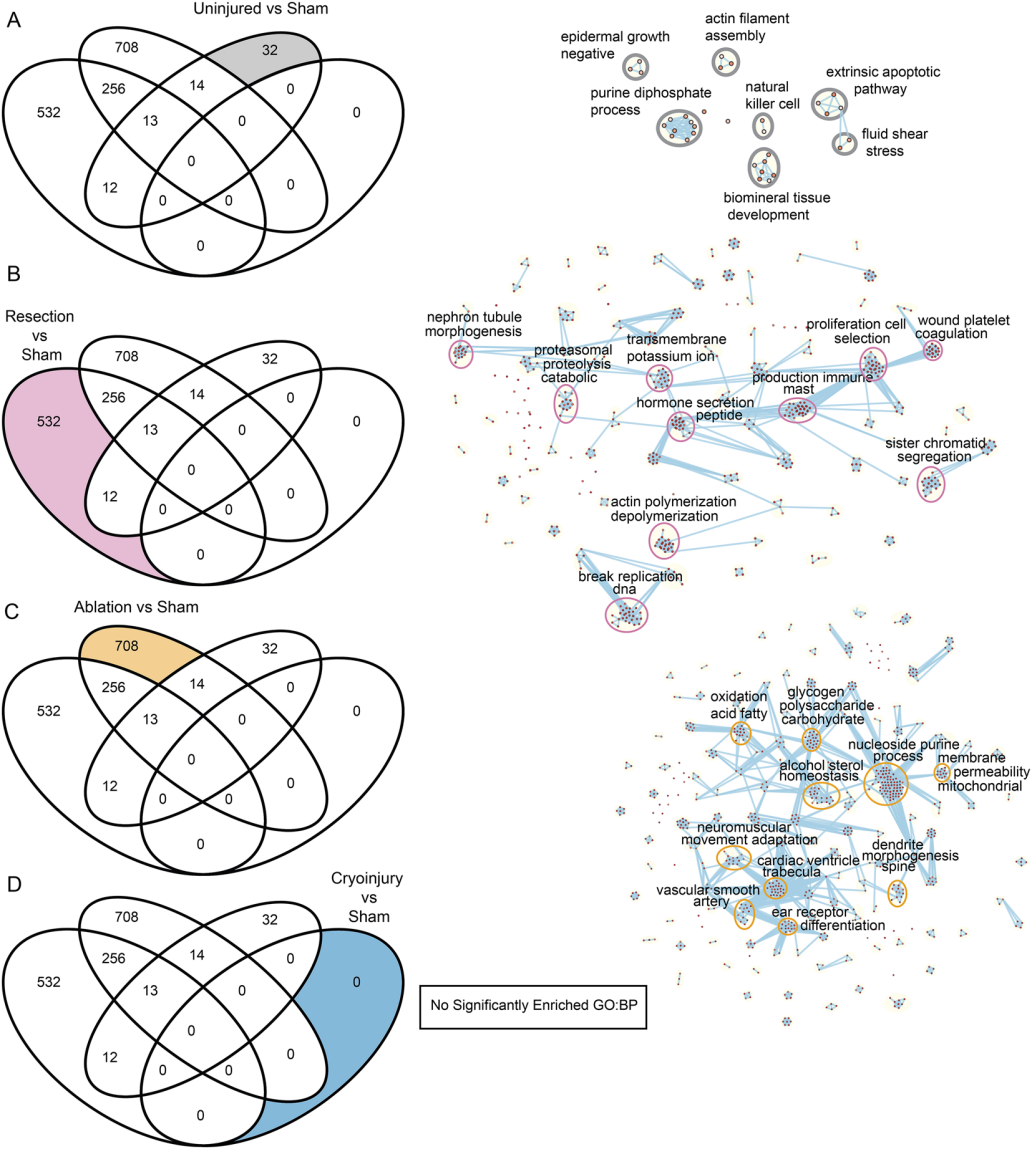
Biological process analysis of DEGs unique to different regeneration models. On the left, Venn diagrams of enriched Gene Ontology Biological Processes showing the specific GO:BP for each condition of interest analyzed labelled in color. On the right, Cytoscape representations of most enriched processes. Shown are data from the comparisons of the different injury models. (**A**) Uninjured vs sham, (**B**) Resection vs sham, (**C**) Ablation vs sham, (**D**) Cryoinjury vs sham, revealing no significantly enriched terms.

The highest number of unique biological processes were found in the comparison ablation vs sham with 708 processes, followed by the identification of 532 processes in resection vs sham, and 32 processes in uninjured vs sham comparisons (Fig. 3).

Gene Ontology terms can be redundant making them difficult to interpret^23,24^. To solve this problem, we decided to group the processes. To this end, we exported the enriched unique GO:BP terms to EnrichmentMap^25^ in Cytoscape^26^ (Fig. 3). This tool uses the GO database as a reference and connects the selected GO:BP by the similarity coefficient (shared genes) between GO:BPs. Then, it returns a network based on genes within the respective GO:BP. The network is then annotated to various clusters using the AutoAnnotate^27^ plugin in Cytoscape, which is based on the similarity coefficient obtained from the EnrichmentMap. Finally, AutoAnnotate tags each of the obtained cluster with the most repeated words from the nodes, i.e., the GO:BP, and forms a word cloud. Thus, each cluster represents a similar set of biological processes. We plotted the network clusters separately for each of the pairwise comparisons and marked the top 10 clusters with the greatest number of nodes.

First, we analyzed the results from the pathways encoded by the DEGs from the uninjured vs sham comparison. We found a total of 32 specific unique biological processes and identified a total of 7 clusters with terms associated with natural killer cell response, apoptosis, epidermal growth factor signaling, purine diphosphate metabolism, actin filament assembly, bone development and fluid shear stress. Thus, even a sham-operation can lead to a significant response that also influences the cardiac gene expression profile and might have physiological consequences (Fig. 3A).

Within the 532 unique GO:BP Terms for resection vs sham, we saw that our enrichment analysis captured the clusters for processes involving wound healing and platelet coagulation. Since resection involves a wound being inflicted to the heart, our analysis confirms its ability to identify processes unique to this injury type. We further found enrichment for processes associated with the immune system (including mast cells), indicative of possible differences in the immune response upon resection compared to the rest of injury models. We also identified processes involved with DNA replication, chromatid segregation and cell proliferation, which are all processes related to tissue regeneration. Also unique to the resection model were processes involved in actin polymerization and depolymerization. We also identified terms associated with cell secretion, hormone secretion and transmembrane ion transports highlighting extracellular communication that might be specific to a resected heart (Fig. 3B).

For the 708 unique GO:BP terms of ablation vs sham, the network clusters displayed terms associated with cardiac trabeculation in the ventricle. Indeed, this corresponds to the predominantly ablated cell population, trabecular cardiomyocytes. We further observed an enrichment of the processes which are associated with the vasculature, neuromuscular adaptations, and morphogenesis. Importantly, metabolic pathways including sterol homeostasis, fatty acid oxidation, purine, and nucleoside processing, as well as carbohydrate and polysaccharide metabolism were among the processes specific to the cardiomyocyte ablation model. Given the importance of a metabolic switch during myocardial regeneration^28^, observing these specific changes in GO:BP might highlight specific metabolic adaptations to the response upon genetic ablation (Fig. 3C).

Interestingly, we did not find biological processes unique to the cryoinjury model (Fig. 3D). It is important to note that this does not mean that there were no differentially expressed genes associated with cryoinjury vs sham, but rather suggests that we could not detect biological processes associated specifically to cryoinjury.

### Identification of core regeneration biological pathways.

**Figure 4:**
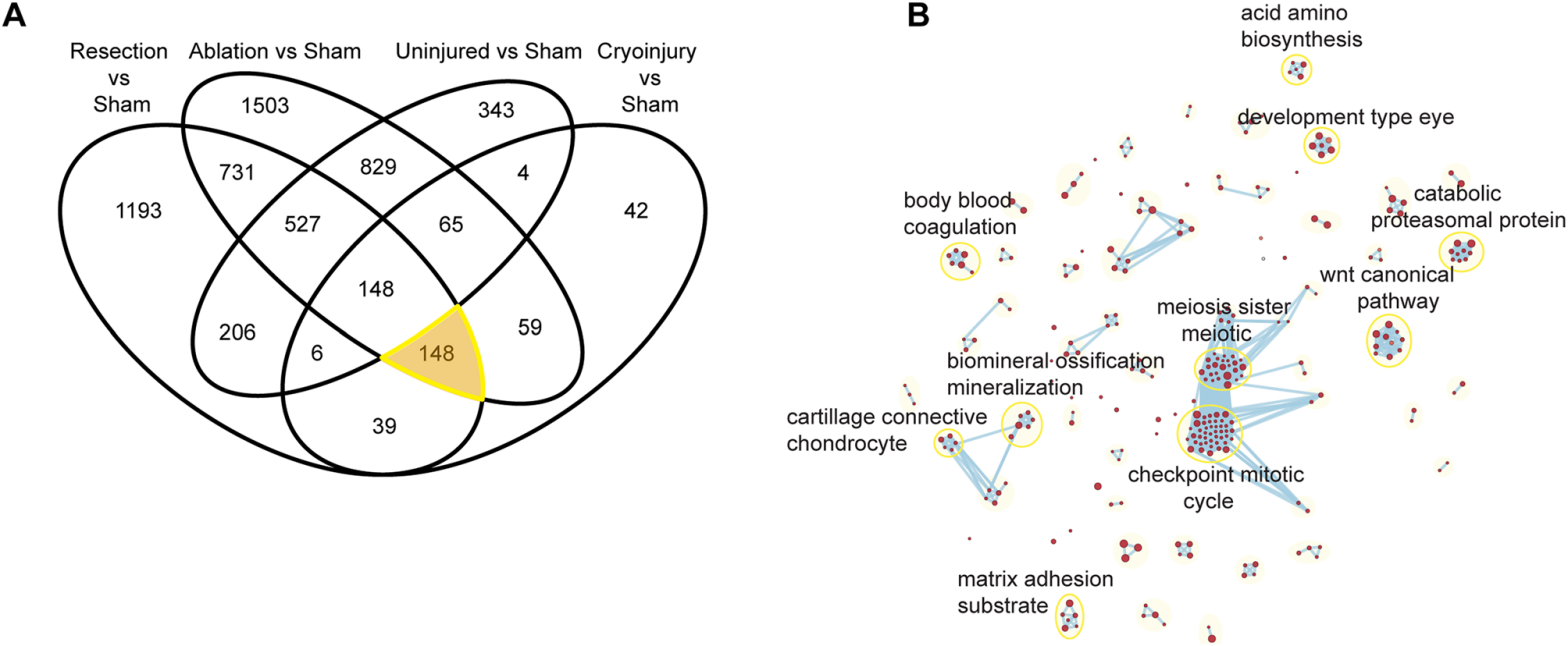
Biological pathways enriched in the core regeneration DEG set. (**A**) Venn diagram of the *Mus musculus* converted genes from the DEG analysis. Highlighting the converted genes involved in the “core regeneration” process of a heart injury despite the model used. (**B**) Gene Ontology Biological Processes associated to these genes when performing annotation and the clustering of these in a network.

To identify common regenerative biological signatures, we performed GO:BP enrichment followed by EnrichmentMap and AutoAnnotate network clustering from the n = 148 into mouse ortholog-converted DEGs which were common across the injury models (Fig. 4A,B and Supplementary Table S3 and Table S4). Of these a majority - 133 genes - are upregulated across injury conditions, with 5 being downregulated and 10 showed mixed response across injuries. Again, we marked the top 10 clusters that were found in the core regeneration.

As expected, cell division related clusters revealed a conserved cell proliferation program across injury conditions. Within this cluster we found, as an example, Aurora kinase B (*Aurkb*), a well-established marker for proliferating cardiomyocytes during cardiomyocyte cell division^29^.

Further, we observed cartilage and bone ossification processes as a core regeneration pathway. Some of the genes that are enriched in these pathways are indeed associated with regeneration across various tissues. Several of the genes within this pathway have been shown to be expressed in fibroblasts of the regenerating zebrafish heart^30,31^. Fibronectin 1 (*Fn1*) for example, is expressed in epicardial derived fibroblasts and plays a role in heart regeneration^32^. Lysyl oxidase (*Lox)*, enriched in cartilage and bone ossification processes, has previously been associated with muscle and cartilage regeneration processes^33,34^. A second gene represented in this pathway is Collagen triple helix repeat containing 1 (*Cthrc1)*. This gene is required within cardiac fibroblasts avoiding lethality following a MI in the mouse^35^. Another fibroblast gene enriched in this pathway that has recently been shown to restrict fibrosis and promote cardiomyocyte proliferation in zebrafish heart regeneration is Paired Related Homeobox 1 (*Prrx1)*^36^. Indeed, when performing an enrichment analysis across databases (using Enrichr)^37,38^ for *core regeneration* genes, fibroblasts come up in the top enriched cell type population in single-cell cell type classification databases- PanglaoDB, Tabula Sapiens, Azimuth Cell Types, ARCHS4 Tissues and HuBMAP^39–43^.

Blood coagulation was another common biological pathway. Within this group, the role of Complement Component 3 (*C3*) has been studied in the context of spinal injury regeneration and is important to preserve myocardial function following a MI^44,45^. Another gene in this group is Heparin-binding EGF-like growth factor (*Hbegf*)*. Hbegf* has been shown to activate non-cardiomyocytes and is involved in cardiac remodeling upon MI^46^.

Metabolic processes are among those pathways strongly represented. We observed metabolic processes involved in amino acids biosynthesis within the core processes shared by all injury conditions. One of the genes within this process is Methylenetetrahydrofolate Dehydrogenase (NADP + Dependent) 1 Like (*Mthfd1l),* which has previously been shown to regulate pathological hypertrophy in the mouse, as it controls endoreplication in this species. In the zebrafish, capable of cytokinesis, *Mthfd1l* orthologs are indeed expected to be pro-regenerative^47^.

Developmental pathways such as the Wnt signaling pathway are reactivated across injury conditions. Indeed, multiple studies have shown the role of Wnt signaling in the context of heart regeneration^48–52^.

Importantly, within the core regeneration genes we were also able to identify genes not previously associated with regeneration. 66 out of the 148 genes (~ 45%) found in our meta-analysis were not associated previously with regeneration (Supplementary Table S4).

In summary, our analysis identified a set of genes with a conserved expression throughout the most widely used experimental injury models. These genes might represent a core signature of a regenerative response. Some of these genes were previously described in the regeneration processes or have been implicated in MI or cardiac diseases, while others represent genes whose roles in heart regeneration have yet to be identified. 

### Visualization of results via R shiny based web app

To help visualize and make all the findings accessible we built a R Shiny based app https://mybinder.org/v2/gh/MercaderLabAnatomy/PUB_Botos_et_al_2022_shinyapp_binder/HEAD?urlpath=shiny/bus-dashboard/. The app aids in visualizing the enriched pathway for each of the compared conditions (Supplementary Movie S1). Each comparison has a different tab. The network of pathways described previously can be accessed in these tabs and the drop-down menu allows access to the broader clusters which group together based on the genes that are shared across the pathways. Each of the enriched genes can be accessed by clicking on the network nodes (each node representing an enriched pathway) and the expression pattern of the enriched genes be visualized as heatmaps. For example, selecting “Canonical Wnt signaling” in “Resection vs Sham” tab brings to focus the four Gene ontology enrichment nodes that are related to Wnt signaling. Selecting one of these nodes “GO:0030177” then brings the enriched genes in the form of a heatmap. Here, we can visualize that *Fgfr3, Lrrk2, Arntl, Atp6ap2, Ttc21b, Dixdc1, Kank1, Zranb1, Shh, Eda, Rnf146, Cav1, Wnk2* are enriched and the corresponding heatmap shows that *Cav1* is most differential amongst these genes.

The app further explores the genes that are previously described in the context of heart regeneration in our PubMed query and the genes that are not described yet. Since the core regeneration genes bring about the potential novel targets, we also show in a tab the log2 fold change of the core regeneration genes in comparison in different injury conditions. Finally in the Gene seeker tab, we show the expression value of genes across all the conditions making the app available to probe for all the genes irrespective of its differential expression status.

In summary we present here an app to make the data more accessible and help in visualization and interpretation.

## Discussion

Despite the simultaneous and apparent interchangeable use within the scientific community of the herein analyzed three injury models it is not fully understood to which extent regenerative responses are equal. Here we describe for one specific time point similarities but also injury-specific gene signatures. To this end, we performed a meta-analysis to compare the transcriptomic profile of regenerating zebrafish hearts using the three main heart injury models. We developed and used a standardized analysis pipeline starting with raw data input up to pathway identification. The raw data without processing revealed a large variability due to batch effects. Such batches have been shown to affect the conclusions including observation of false positives and false negatives, leading to misinterpretations^53–55^. In fact, batch correction has led to improvement in identification of genes which are differentially expressed and improved the existing datasets for the comparisons across experiments^56^. In our study, we used ComBat-Seq for batch correction. While we expected that experimental differences such as collection of whole hearts or cardiac ventricles would introduce the highest variability, we observed the correction for the used sequencing platforms to be the most relevant variable.

When we next performed the differential expression analysis, we decided to use DESeq2 and edgeR algorithms in parallel. Both tools follow similar hypotheses that no genes are differential but use different methods for normalization. The normalization process is followed by a statistical testing to determine differentially expressed genes. In our analysis, we found DESeq2 to be more stringent and to retrieve a subset of the genes also found by edgeR and therefore used these results for our RNA-seq meta-analysis. With our analysis we found genes which are injury specific and genes that form the core regeneration genes. Our unbiased pipeline and strict thresholds ensure minimum false positives and help differentiate between different responses. We observed most differentially expressed genes in the ablation vs sham, the response also had the greatest number of unique genes (genes not observed in other injury types). These results confirm the observation that ablation with no fibrosis elicits a different response than resection or cryoinjury.

To get further biological insights into the functional aspects of the identified genes, zebrafish genes passing the filtering threshold were translated to their corresponding *Mus musculus* orthologs. The reason for this conversion is that the annotation of *Mus musculus* translated terms returns a broader set of biological information. It is worth noting that multiple zebrafish paralogues correspond to one orthologue in *Mus musculus*. Though many of the genes have an annotated orthologue, not all zebrafish genes could be converted into mouse orthologs, presenting a limitation of the study. We do however strongly recommend such a conversion when analyzing zebrafish transcriptomes, at least until GO annotation becomes more comprehensive also for this species.

Our analysis also allowed us to identify biological pathways that were injury specific. Ventricular resection is based on tissue removal and elicits a blood coagulation response. In line with our GO:BP analysis we observed biological pathways related to wound healing to be specific to this type of injury. Genetic ablation led to the highest amount of specific biological pathways uniquely altered. This injury model specifically targets the myocardium. Metabolic changes precede a regenerative response in cardiomyocytes^28,57,58^. Indeed, most of the processes specific to the genetic ablation were associated to metabolism and to cardiomyocytes. During genetic ablation no sham-operation is needed. We therefore also performed the genetic ablation vs uninjured heart comparison without obtaining major differences regarding enriched biological process (data not shown).

Even though ventricular cryoinjury is associated with a fibrotic response that is more significant compared to the other two injury models, we found no statistically significant enriched Biological Processes unique to cryoinjury. On the contrary, this injury model encompasses most of the genes and biological processes also observed in the other injury models or in the core regeneration response.

Our analysis shows that the sham surgery to open the heart cavity induces a systemic inflammatory response. Therefore, we think that sham 7dpi vs uninjured reveals even more DEG that sham 7dpi vs cryoinjury 7 dpi. Indeed from 853 genes from cryoinjury vs sham comparisons, 372 (42% of genes) (Fig. 2B) still overlap with uninjured vs sham. This leads us to suggest that the regeneration program initiated by cryoinjury has the least response triggered by the injury method itself. It must be noted that the time point used was 7dpi for all injury models. It is known that there are temporal differences in the regeneration stage among injury methods. Therefore, some of the apparent differences in gene expression across methods at 7dpi, could reflect the different stage of regeneration.

We also identified a set of genes differentially expressed in all the three heart injury models, which we named *core regeneration genes*. It is somewhat surprising not to identify a larger number of genes within this shared DEG set. It being a relatively small list of genes also suggests that they might be particularly relevant for heart regeneration. Indeed, Wnt receptors and signaling regulators such as *Fzd9, Lrp1, Dkk3* and *Alpk2* are among the *core regeneration genes* and the Wnt signaling pathway is known to be crucial for heart regeneration^30,50,59^. Many *core regeneration* genes (*Fkbp10, Postn, Tagln, Prrx1, Lum, Htra3, Fn1, Dkk3, Lox, Mdk, Ctsk, Cdh11, Col8a1, Itgbl1, Angptl2, Pamr1, Ddr2, Cthrc1*) represent fibroblast gene markers. Fibroblasts mediate Extracellular matrix (ECM) deposition, and the ECM produced during the regeneration is known to be crucial for the cardiomyocyte proliferation and the remodeling of the injured heart^60^. Perturbation of Fibronectin (*Fn1a*) production or ablation of Collagen alpha 2 (I) (*Col1a2*) producing cells leads to defects in cardiomyocyte proliferation in genetic ablation and cryoinjury models^31,32^. Many of the same ECM proteins have also been reported from ECM of resected hearts^61^. Furthermore, ECM from resected zebrafish hearts has been shown to induce proliferation of human myocardiocytes in vitro^62^. Thus, the presence of these ECM genes in our *core regeneration genes* indicate that a fibrotic response is inherent to the regenerative response and also supports functional studies that revealed a role for fibroblasts in heart regeneration^30,31^.

Within the set of core regeneration nearly half of the genes had not been previously studied in the context of regeneration. These included molecules mediating cell–cell adhesion, such as *Nectin1* or cell–matrix adhesion proteins, such as Integrin subunit beta like 1 (*Itgbl1)*. These are also enriched in various cell adhesion and development related GO terms. Other genes were associated with metabolism, e.g. Glucosamine-6-phosphate deaminase (*Gnpda1*). Their functional assessment in the context of regeneration might give new insights into the altered metabolic states of the regenerating hearts. A further interesting set of genes not fully studied during heart regeneration are related to the cytoskeleton. The GTPases and GAPs like *Rap1gap, Rassf6* and *Rnd3* are new potential candidates to explore how actomyosin dynamics control heart regeneration and the study of the microtubule associated serine/threonine kinase 1 (*Mast1*) and microtubule associated serine/threonine kinase like (*Mastl*) might allow to better understand the role of microtubules in this process or in cell division related pathways.

Finally, we made our results accessible via a user-friendly web interface. Through our web-app one can access all injury specific and core regeneration genes. It provides an interface to understand the GO:BP pathways that are enriched in each of these conditions and makes it easy to compare across the conditions. Our interface has interactive network maps for each condition that was analyzed giving the details of the genes enriched in the pathways along with complementary heatmaps showing their expression patterns. We enhance these functionalities across datasets with the Gene Seeker functionality that will help users understand the expression pattern of each gene in each condition across datasets. While there exist other online portals that document cardiac regeneration processes none of the studies provide a solution that involves various injury conditions nor provide batch corrected normalized comparable data^63,64^. Further, some of the studies focus on non-coding RNAs, or utilize older data such as from microarray^65^. We present the first portal allowing the comparison of gene expression changes in different injury models. This interface represents a valuable tool to study genes and pathways, specific or common to the process of heart regeneration among different injury responses.

Overall, the presented re-analysis of RNA-seq data allowed the identification of a group of pan-regenerative genes and at the same time define injury-specific gene expression programs. The results were obtained without the need of additional animal experiments highlighting the importance of transcriptomic meta-analysis for the contribution to the 3R principles.

## Materials and methods

### Pre-processing and quality filtering

Raw data were downloaded in fastq format from GEO (https://www.ncbi.nlm.nih.gov/geo) using sratoolkit 2.10.7^66^. Quality check was performed using FastQC 0.11.7^67^ on each sample, pre and post trimming. Trimming was done for cleaning adapters and low-quality reads using fastp 0.20.1^68^. The trimmed reads that passed the filters were aligned to *Danio rerio* reference genome GRCz11^69^ from Ensembl and the GTF file from Ensembl version 102^70^ using STAR 2.7.3a^71^. In STAR, the ”*genecounts”* argument was used in star to directly quantify the read counts.

### Batch correction/integration

We used Combat-seq available through the package sva 3.4.0^72^, to remove the batch effect in the gene expression data. Combatseq is a RNA-seq tailored batch correction tool, it uses a negative binomial regression that models the bulk RNA-seq count data and statistically adjusts the model with the expression values in regard of the batch effects and supplies a corrected matrix of counts that can be directly fed for differential expression analysis including DESeq2 that requires integer as the input.

### Differential expression analysis and normalization

To identify differentially expressed genes between heart injury models, we used DESeq2 package 1.32.0. We normalized the data using the default *DESeq* function. We then performed the pairwise comparisons where log fold changes were shrunk with the *lfcShrink* function using “ashr” shrinkage *method (*version 2.2-47) for a precise log2 fold change variance calculation^73^. The *p* values were calculated using the Wald significance test on the library size and library composition normalized gene expression values. These *p* values were then adjusted for multiple testing using Benjamini and Hochberg correction. For edgeR, we used the TMM normalization method and then performed differential expression analysis on the same datasets as used in the DESeq2 workflow. Only genes with an adjusted *p* value, less than 0.05 and an absolute value of the log2 fold change greater than 1 were kept as differentially expressed. The differential gene lists were later used for an over-representation analysis.

### Over-representation analysis

The gene list associated to each contrast was subjected to Gene Ontology enrichment analysis via the R package clusterProfiler 4.0.5. For improved annotations, the zebrafish genes were converted to *Mus musculus* orthologs, using biomaRt package 2.48.3^74^ and selecting the gene with the highest homology percentage parameter. For 52% of the zebrafish genes, we were able to find mouse orthologues. Entrez IDs that function as an input for the clusterProfiler, were obtained using biomaRt (Supplementary Table S2). We then filtered the genes and selected the genes unique to each pairwise comparison. For the core regeneration genes, the genes were filtered for common genes amongst all the pairwise comparison, except uninjured vs sham. To perform the over representation analysis on these filtered genes, clusterProfiler:enrichGO was used to access the Gene Ontology (GO) database and selecting the Biological Processes (BP) terms as annotation. We used the threshold of terms with adjusted *p* value < 0.05 to select the significantly enriched processes. Visualization of the enriched terms was performed via customized R language functions using ggplot2^75^ and Venn Diagram^76^.

### Network visualization of GO:BP terms

We filtered the terms obtained from overrepresented analysis to get unique terms for each of the pairwise comparisons. This allowed to remove the terms that might overlap across comparisons. These filtered terms were exported to Cytoscape 3.9.1, where EnrichmentMap 3.3.3 plugin was used to build a network of the significantly enriched GO:BP terms. Using EnrichmentMap, we could group the GO:BP terms by the similarity coefficient, being this the number of shared genes in the GO:BP associated terms. After generating the GO:BP network, we used AutoAnnotate 1.3.5 to label and group the network modules using the arguments "*MCL*” and “*similarity coefficient*”.

### PubMed query

For the query, each individual gene found in the core regeneration was submitted followed by the search term “regeneration” to PubMed using the R easyPubMed package^77^. If the combination of gene AND regeneration was found in the title or abstract of a paper the PubMed Id of the paper was retrieved, the corresponding gene was included as previously studied in the context of regeneration.

## Supplementary Information

Supplementary Figure S1.

Supplementary Table S1.

Supplementary Table S2.

Supplementary Table S3.

Supplementary Table S4.

Supplementary Video 1.

Supplementary Legends.

## Data Availability

For reproducibility and access to the data used in this analysis the samples used can be accessed through their direct link to GSE in Table 1. For the analysis workflow used the code is available through in the repository https://github.com/MercaderLabAnatomy/PUB_Botos_et_al_2022/. Furthermore, we also made all the processed data available through a shinyR based web application hosted at using Binder 2.0 for reproducible interactive sharable environments at mybinder.org^78^ at the link- https://mybinder.org/v2/gh/MercaderLabAnatomy/PUB_Botos_et_al_2022_shinyapp_binder/HEAD?urlpath=shiny/bus-dashboard/. Through the app we show the clustered networks and the genes and their expression levels in each of the biological process.
