# Correction: Transcriptomic data meta-analysis reveals common and injury model specific gene expression changes in the regenerating zebrafish heart

**DOI:** 10.1038/s41598-026-50693-x

**Published:** 2026-05-01

**Authors:** Marius Alexandru Botos, Prateek Arora, Panagiotis Chouvardas, Nadia Mercader

**Affiliations:** 1 Institute of Anatomy, University of Bern, 3012 Bern, Switzerland; 2 Department for Biomedical Research, University of Bern, 3012 Bern, Switzerland; 3 Department of Urology, Inselspital, Bern University Hospital, 3010 Bern, Switzerland; 4 Centro Nacional de Investigaciones Cardiovasculares CNIC, 28029 Madrid, Spain; 5 Present Address: Laboratory of Systems Biology and Genetics, Institute of Bioengineering, School of Life Sciences, École Polytechnique Fédérale de Lausanne (EPFL), Lausanne, Switzerland; 6 Present Address: Swiss Institute of Bioinformatics (SIB), Lausanne, Switzerland

Correction to: *Scientific Reports* 10.1038/s41598-023-32272-6, published online 03 April 2023

The original version of this Article contains errors. Due to a labeling error, three samples with ‘Resection’ status under accession number GSE144831 were inadvertently misclassified as ‘Uninjured’. Consequently, the sample sizes for both the ‘Uninjured’ and ‘Resection’ cohorts were affected, resulting in errors in Figures 1–4, in the Supplementary Figure S1 and in the Supplementary Table S1–4 files. The scientific conclusions of the study remain unaffected.

The correct Figures 1, 2, 3, 4 and accompanying legends appear below.

**Fig. 1:**
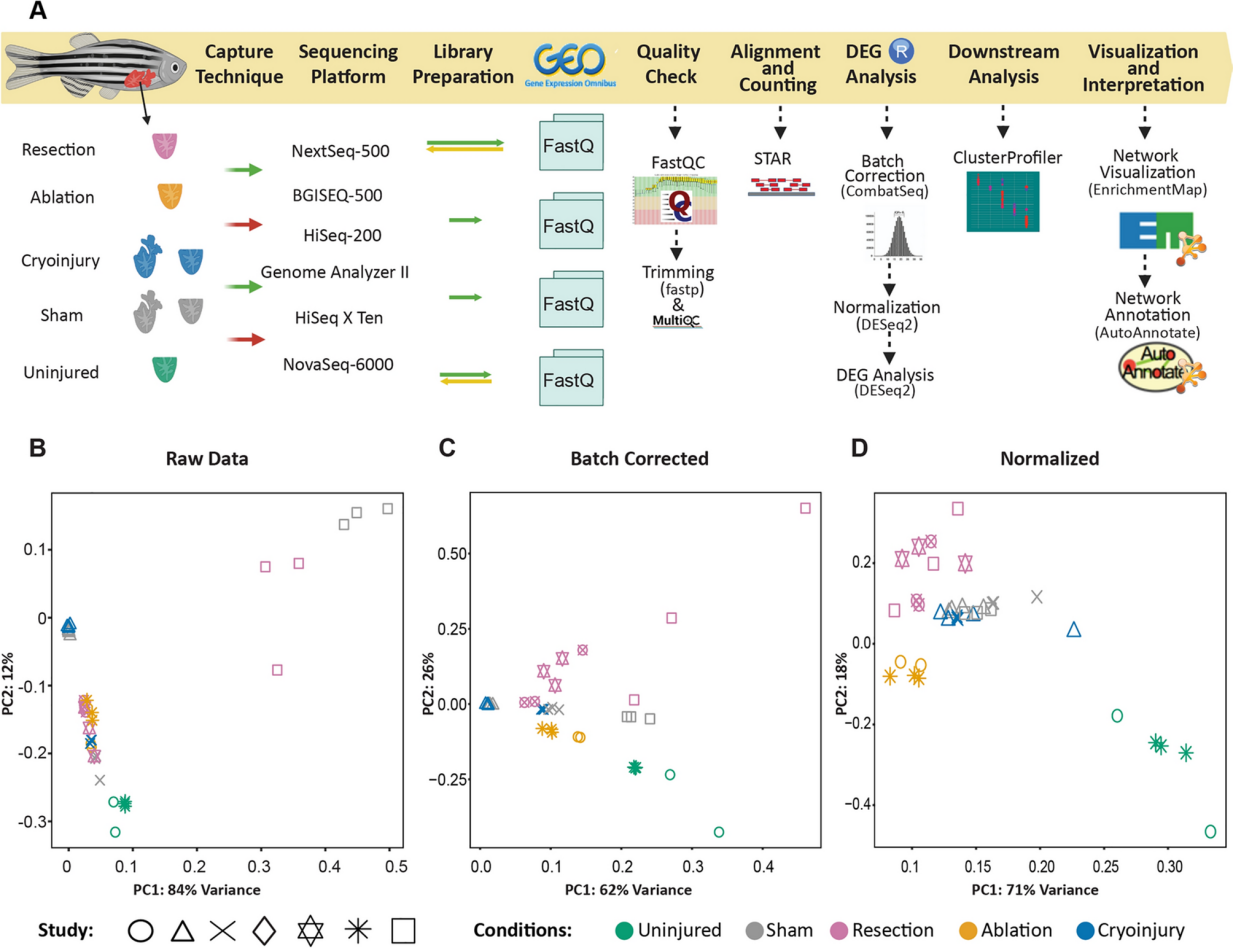
Transcriptomic meta-analysis of the cardiac regenerative response upon distinct types of injuries. (**A**) Workflow of the process used to analyze the data. Selected datasets differed in conditions of the transcriptomic data such as the capture techniques, sequencing platforms and reads structure. Data were downloaded from GEO and the processing steps to find the differentially expressed genes were performed. This was followed by downstream analysis to interpret the results. (**B**) PCA plots of the raw data before any processing. (**C**) Batch corrected, after removing the platform sequencing variable giving the highest batch effect. (**D**) DESeq2 normalized data.

**Fig. 2:**
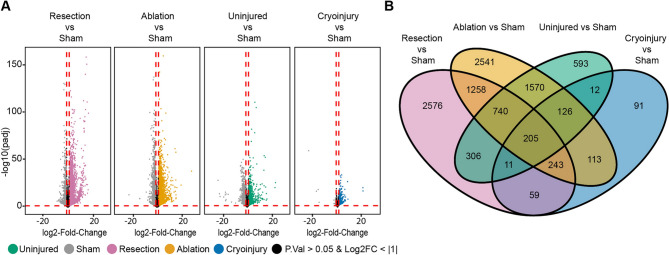
Unique and common differentially expressed genes (DEGs) in the 7 dpi regenerating zebrafish heart after resection, cryoinjury, or genetic ablation. (**A**) Volcano plot of the different conditions showing the DEGs. Grey, sham; orange, genetic ablation; magenta, ventricular resection; green, uninjured; blue, cryoinjury. Black area stands for non-significant genes with an adjusted *p* value > 0.05 or a log2FoldChange value > − 1 and <  + 1 which were not considered for the analysis. (**B**) Venn diagram of the zebrafish DEGs converted to the respective mouse orthologs, for each condition.

**Fig. 3:**
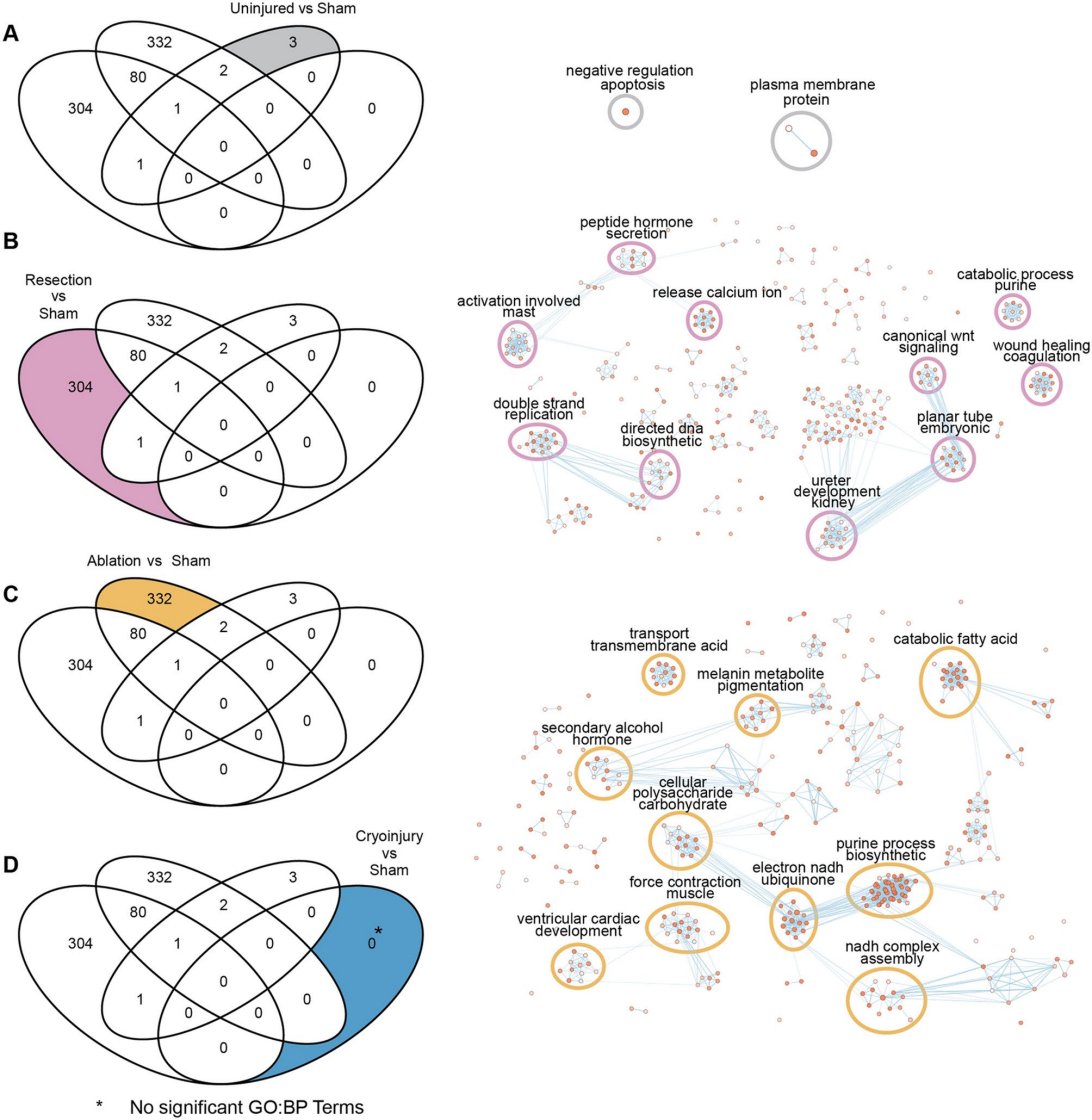
Biological process analysis of DEGs unique to different regeneration models. On the left, Venn diagrams of enriched Gene Ontology Biological Processes showing the specific GO:BP for each condition of interest analyzed labelled in color. On the right, Cytoscape representations of most enriched processes. Shown are data from the comparisons of the different injury models. (**A**) Uninjured vs sham, (**B**) Resection vs sham, (**C**) Ablation vs sham, (**D**) Cryoinjury vs sham, revealing no significantly enriched terms.

**Fig. 4:**
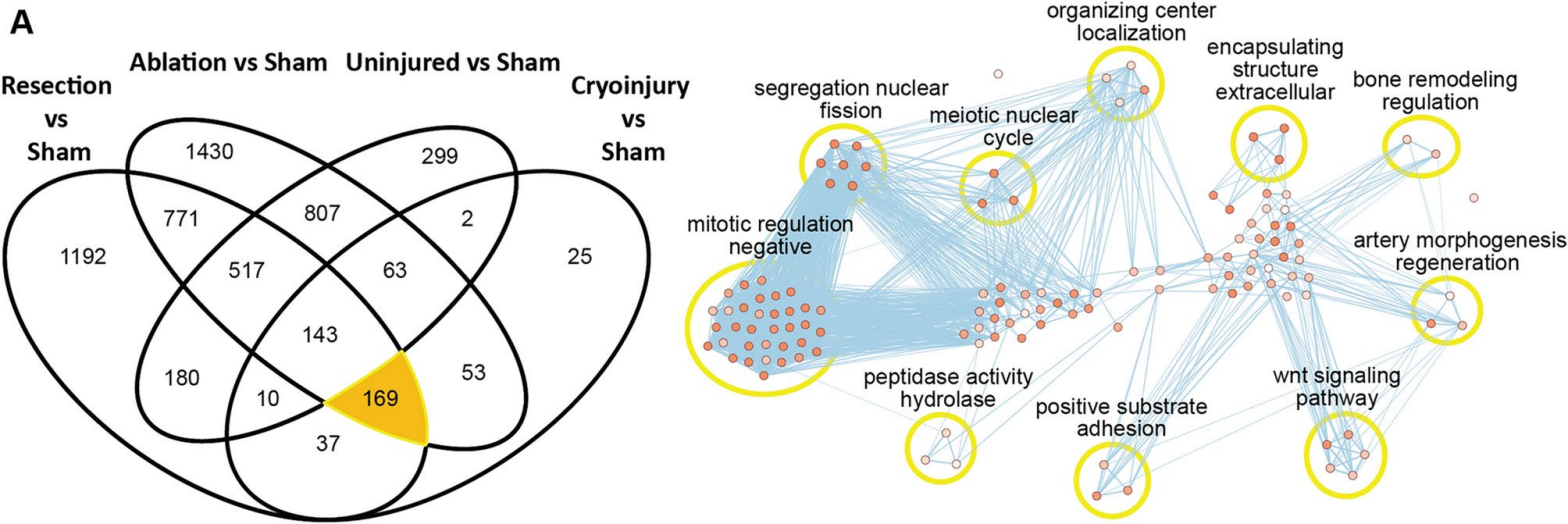
Biological pathways enriched in the core regeneration DEG set. (**A**) Venn diagram of the *Mus musculus* converted genes from the DEG analysis. Highlighting the converted genes involved in the “core regeneration” process of a heart injury despite the model used. (**B**) Gene Ontology Biological Processes associated to these genes when performing annotation and the clustering of these in a network.

The correct Supplementary Information files are now linked to this correction notice.

## Supplementary Information

Supplementary Figure S1.

Supplementary Table S1.

Supplementary Table S2.

Supplementary Table S3.

Supplementary Table S4.

