# Supplementary figures and images for: Transcriptomic data meta-analysis reveals common and injury model specific gene expression changes in the regenerating zebrafish heart
Source: Sci Rep. 2023 Apr 3;13:5418. doi: 10.1038/s41598-023-32272-6 (PMC10070245; doi:10.1038/s41598-023-32272-6)

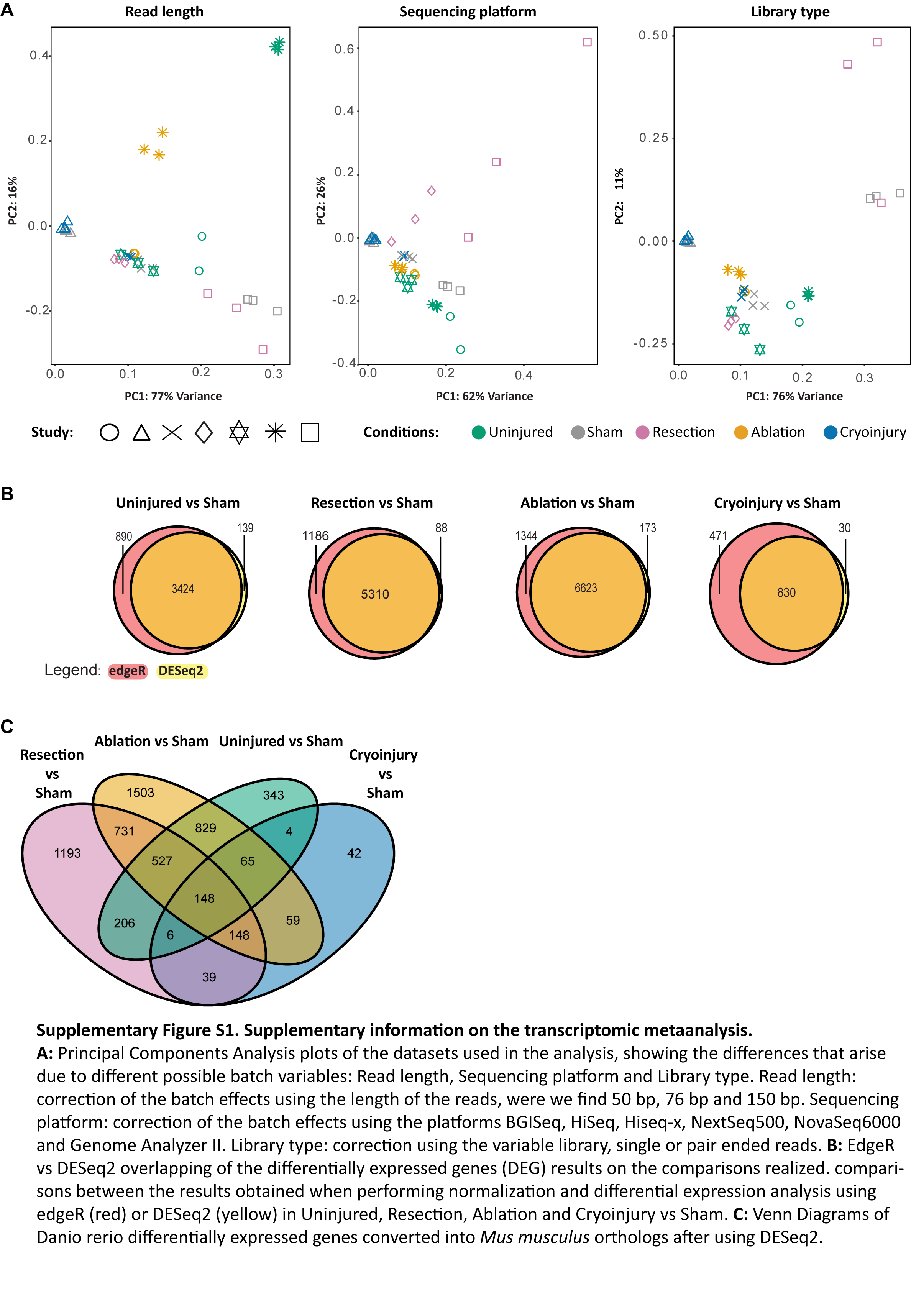

Supplement: Supplementary file 1 — Supplementary Figure S1. [file 41598_2023_32272_MOESM1_ESM.tif]
