# Supplementary material for: Transcriptomic data meta-analysis reveals common and injury model specific gene expression changes in the regenerating zebrafish heart
Source: Sci Rep. 2023 Apr 3;13:5418. doi: 10.1038/s41598-023-32272-6 (PMC10070245; doi:10.1038/s41598-023-32272-6)
Supplement: Supplementary file 7 — Supplementary Legends. [file 41598_2023_32272_MOESM7_ESM.docx]

**Supplementary Data Legends**

**Supplementary Figure S1. Supplementary information on the transcriptomic metaanalysis.**

**A:** Principal Components Analysis plots of the datasets used in the analysis, showing the differences that arise due to different possible batch variables: Read length, Sequencing platform and Library type. Read length: correction of the batch effects using the length of the reads, were we find 50 bp, 76 bp and 150 bp. Sequencing platform: correction of the batch effects using the platforms BGISeq, HiSeq, Hiseq-x, NextSeq500, NovaSeq6000 and Genome Analyzer II. Library type: correction using the variable library, single or pair ended reads. **B:** EdgeR vs DESeq2 overlapping of the differentially expressed genes (DEG) results on the comparisons realized. comparisons between the results obtained when performing normalization and differential expression analysis using edgeR (red) or DESeq2 (yellow) in Uninjured, Resection, Ablation and Cryoinjury vs Sham. **C**: Venn Diagrams of *Danio rerio* differentially expressed genes converted into *Mus musculus* orthologs after using DESeq2.

**Supplementary Table 1. Statistical pairwise comparisons using DESeq2 or edgeR.**

Shown in the table are the statistical results of the comparisons, including, and identifier such as the respective ensembl gene id, base mean, p-values, adjusted p-values and log-fold-change columns for each respective comparison and tool used.

**Supplementary Table 2. Translation of the *Danio rerio* Ensembl gene id terms to Mus musculus.** Shown are the ensembl gene ids of *Danio rerio* converted to *Mus musculus* after the translation process using biomaRt, associated in the table are also found the statistical information and the batch corrected and normalized counts of each gene for each sample.

**Supplementary Table 3. Downstream analysis of the DEG translated to *Mus musculus* terms and used for Gene Ontologies Biological Processes over representation analysis.** Finding all the comparisons realized with their respective enriched GO: BP.

**Supplementary Table 4. *Mus musculus* translated genes list, common to the three injury models.** PubMed query using the term regeneration AND the respective *core regeneration gene*. Shown are the results of the literature search performed on 18.08.2022. Gene appearing in the literature in the context of regeneration are shown in green, those for which no match with the keyword was obtained are marked in red.

**Supplementary Movie 1.** Tutorial of the Shiny App for mining the transcriptomic data meta-analysis.
