# Supplementary figures and images for: Correction: Transcriptomic data meta-analysis reveals common and injury model specific gene expression changes in the regenerating zebrafish heart
Source: Sci Rep. 2026 May 1;16:14031. doi: 10.1038/s41598-026-50693-x (PMC13134963; doi:10.1038/s41598-026-50693-x)

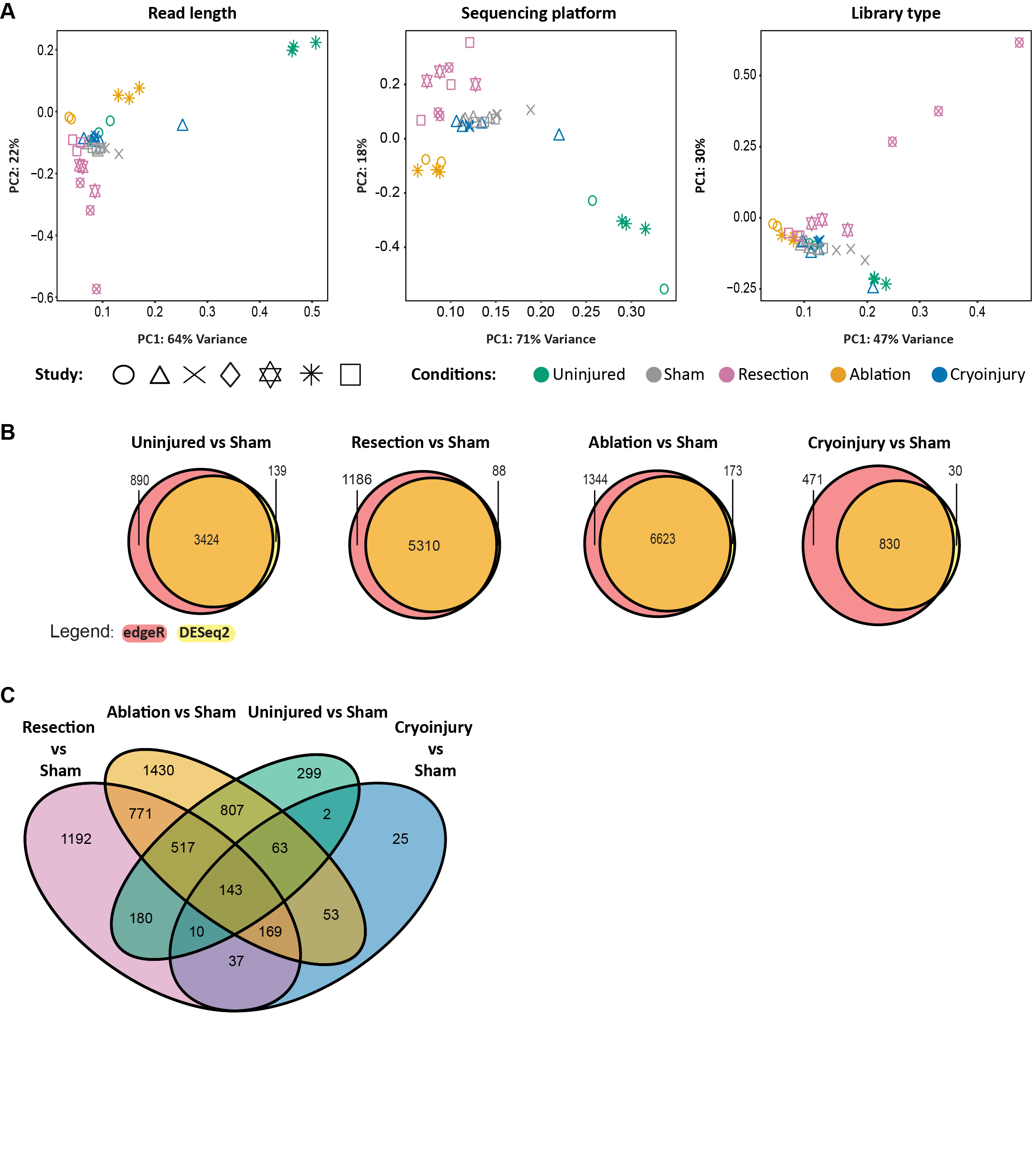

Supplement: Supplementary file 1 — Supplementary Figure S1. [file 41598_2026_50693_MOESM1_ESM.tif]
